# Supplementary material for: The Association between Dynamic Changes in Serum Presepsin Levels and Mortality in Immunocompromised Patients with Sepsis: A Prospective Cohort Study
Source: Diagnostics (Basel). 2021 Jan 2;11(1):60. doi: 10.3390/diagnostics11010060 (PMC7823693; doi:10.3390/diagnostics11010060)
Supplement: Supplementary file 1 [file diagnostics-11-00060-s001.zip › Supplementary Table 1.docx]

**Supplementary Table 1.** Clinical and laboratory characteristics of immunocompromised patients with sepsis

|  | Survived patient  (n = 18) | Died patient  (n = 23) | P value |
| --- | --- | --- | --- |
| Sex, male | 10 (55.6) | 8 (34.8) | 0.311 |
| Age, yrs | 64.9 ± 11.0 | 59.0 ± 14.0 | 0.148 |
| Charlson comorbidity index | 6.0 (5.0 – 9.0) | 4.0 (3.0 – 7.0) | 0.109 |
| SAPS3 score | 78.2 ± 9.5 | 82.9 ± 13.3 | 0.215 |
| SOFA score | 9.8 ± 3.1 | 10.4 ± 3.0 | 0.490 |
| Use of vasopressor on Day 1 | 15 (83.3) | 14 (60.9) | 0.221 |
| Use of invasive ventilation on Day 1 | 4 (22.2) | 10 (43.5) | 0.275 |
| Systolic BP at ICU admission | 78.9 ± 13.8 | 86.5 ± 13.5 | 0.085 |
| Diastolic BP at ICU admission | 45.1 ± 11.2 | 52.2 ± 12.6 | 0.067 |
| Heart rate at ICU admission | 133.7 ± 26.7 | 139.0 ± 25.6 | 0.520 |
| Respiratory rate at ICU admission | 31.5 (29.0 – 35.0) | 31.0 (27.5 – 36.5) | 0.854 |
| Leukocyte count, Day 1 | 3.9 (0.9 – 11.4) | 4.6 (0.6 – 13.2) | 0.834 |
| Neutrophil count, Day 1 | 2.9 (0.3 – 9.0) | 3.4 (0.3 – 10.8) | 0.927 |
| Platelet count, Day 1 | 39.0 (18.0 – 163.0) | 37.0 (24.5 – 77.5) | 0.454 |
| Lactate, Day 1 | 3.3 (2.0 – 7.0) | 2.8 (1.9 – 6.6) | 0.743 |
| Lactate, Day 3 | 17 (1.2 – 2.6) | 2.5 (1.4 – 5.4) | 0.187 |
| Procalcitonin, Day 1 | 25.1 (2.6 – 60.7) | 6.1 (2.4 – 25.4) | 0.156 |
| Procalcitonin, Day 3 | 8.6 (1.3 – 23.4) | 14.1 (2.8 – 41.4) | 0.415 |
| ΔProcalcitonin+ | 2 (11.1) | 10 (43.5) | 0.056 |
| Presepsin, Day 1 | 1892.5 (1490.0 – 3365.0) | 1116.0 (773.0 – 3141.5) | 0.355 |
| Presepsin, Day 3 | 1812.5 (919.0 – 3247.0) | 1449 (987.5 – 3594.0) | 0.590 |
| ΔPresepsin+ | 6 (33.3) | 18 (78.3) | 0.010 |

SAPS3, Simplified Acute Physiology Score 3; SOFA. Sequential Organ Failure Assessment; BP, blood pressure; ICU, intensive care unit; ΔProcalcitonin+, serum procalcitonin level on day 3 minus day 1 > 0; ΔPresepsin+, plasma presepsin level on day 3 minus day 1 > 0.
