# Supplementary figures and images for: The Association between Dynamic Changes in Serum Presepsin Levels and Mortality in Immunocompromised Patients with Sepsis: A Prospective Cohort Study
Source: Diagnostics (Basel). 2021 Jan 2;11(1):60. doi: 10.3390/diagnostics11010060 (PMC7823693; doi:10.3390/diagnostics11010060)

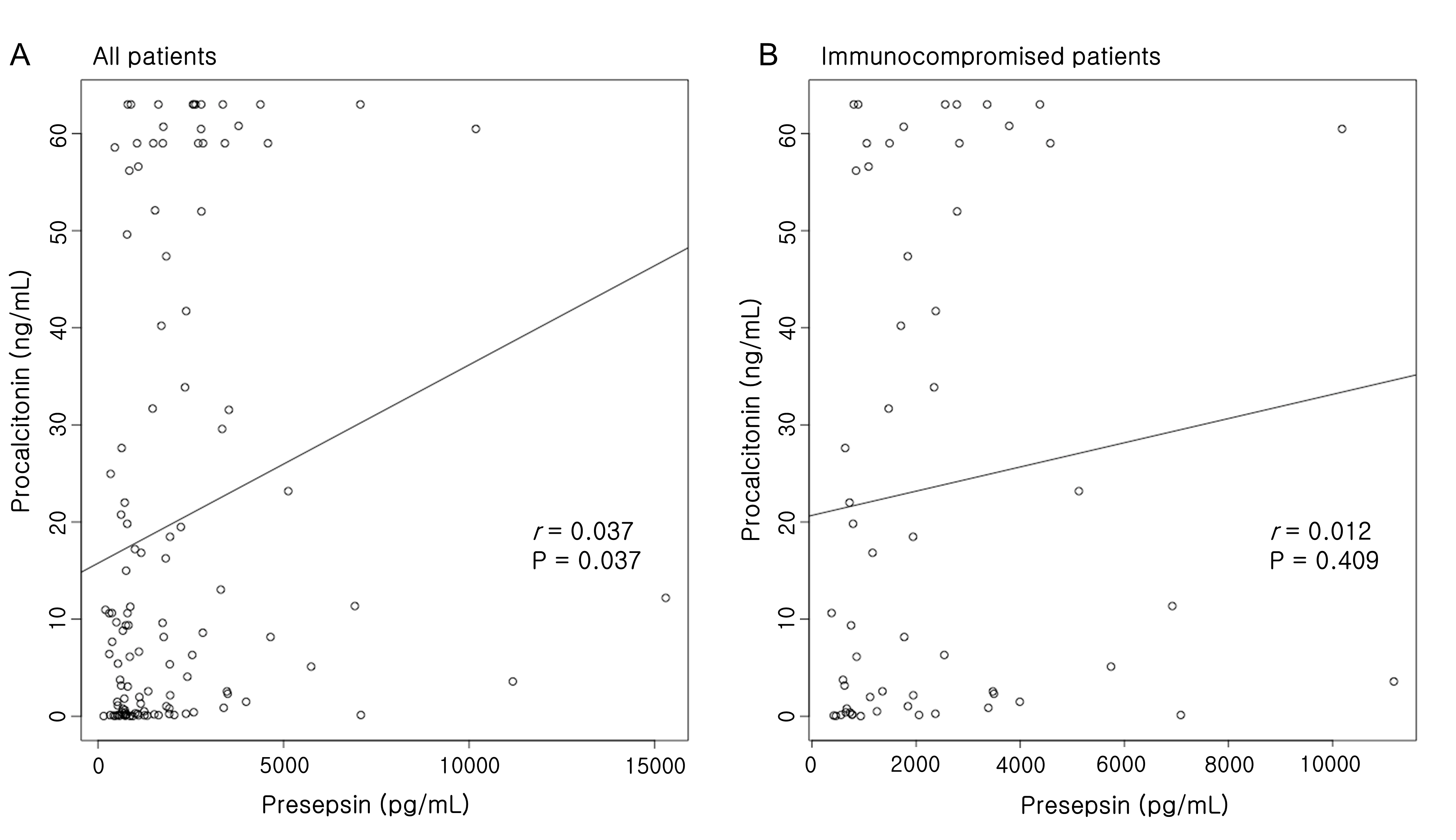

Supplement: Supplementary file 1 [file diagnostics-11-00060-s001.zip › SUP_FIG1_Diagnostics_REVISED.png]
